# Formulation and Evaluation of Galantamine Gel as Drug Reservoir in Transdermal Patch Delivery System

**DOI:** 10.1155/2015/495271

**Published:** 2015-02-26

**Authors:** Woo Fong Yen, Mahiran Basri, Mansor Ahmad, Maznah Ismail

**Affiliations:** ^1^Department of Chemistry, Faculty of Science, Universiti Putra Malaysia, 43300 Serdang, Selangor, Malaysia; ^2^Laboratory of Molecular Biomedicine, Institute of Bioscience, Universiti Putra Malaysia, 43300 Serdang, Selangor, Malaysia

## Abstract

Galantamine hydrobromide is formulated in tablets and capsules prescribed through oral delivery for the treatment of Alzheimer's disease. However, oral delivery of drugs can cause severe side effects such as nausea, vomiting, and gastrointestinal disturbance. Transdermal delivery of galantamine hydrobromide could avoid these unwanted side effects. In this work, galantamine hydrobromide was formulated in gel drug reservoir which was then fabricated in the transdermal patch. The in vitro drug release studies revealed that the drug release from the donor chamber to receptor chamber of Franz diffusion cell was affected by the amount of polymer, amount of neutralizer, amount of drug, types of permeation enhancer, and amount of permeation enhancer. Visual observations of the gels showed that all formulated gels are translucent, homogeneous, smooth, and stable. These gels have pH in the suitable range for skin. The gel also showed high drug content uniformity. Hence, this formulation can be further used in the preparation of transdermal patch drug delivery system.

## 1. Introduction

**Scheme 1:**
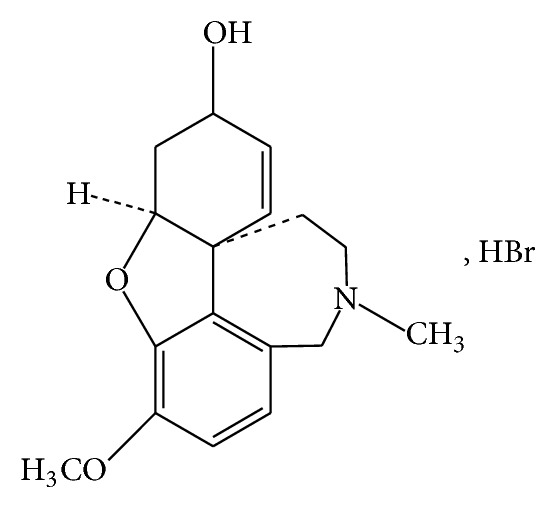
Chemical structure of galantamine hydrobromide [7].

Galantamine hydrobromide is a tertiary alkaloid, which is isolated from various plant species such as* Narcissus* and* Lycoris* species. It is useful in the treatment of Alzheimer's disease by inhibiting the activities of acetylcholinesterase enzyme. This prevents the breakdown of neurotransmitter, acetylcholine. Hence, acetylcholine can survive and keep its function as neurotransmitter, which transmits signals across synapses [1–3]. Galantamine hydrobromide has advantages such as lower muscarinic side effects and higher speed of recovery from respiratory depression, can penetrate through the blood-brain barrier, can bind to nicotinic acetylcholine receptors, and enhanced microglial amyloid-beta peptides phagocytosis [4–6].  Scheme 1 shows the chemical structure of galantamine hydrobromide [7].

Transdermal patch is a system that delivers drugs to the targeted cells or organs by passing through the human biggest organ, skin. The drug molecules will then enter the blood circulation system and then the targeted sites. Transdermal patch drug delivery provides many advantages. Firstly, drug-loaded patch allows longer treatment time for diseases. Thus, patients need less frequent dosing compared to other drug administration methods. Transdermal drug delivery contributes to lesser side effects such as nausea, vomiting, and gastrointestinal disturbance. Patch can also be removed easily and immediately if any side effects are detected. It is also patient friendly especially for unconscious patient, nauseated patient, and the patient with swallowing difficulties [8].

There were some requirements for the compositions used in the gel drug reservoir for transdermal patch. The polymer used for the preparation of gel drug reservoir must be stable, compatible with drugs and other components in gel, inexpensive, and easily fabricated into desired products and provides effective controlled drug release. The penetration enhancers should have reversible impact on skin, not irritating, nontoxic, compatible with formulation, odourless, and colourless. For drug molecules used in gel drug reservoir, they should have low molecular weight (less than 1000 g/mol), low partition coefficient (log⁡*P* less than 4), half-life of less than 10 h, low oral bioavailability, and adequate solubility in their carrier and should not be irritating to body tissues [9, 10].

Carbopol is a synthetic, highly cross-linked, and hydrophilic polymer [11, 12]. It produces transparent and smooth gel when its concentration is above 0.50% [12]. The addition of the triethanolamine to polymer solution can neutralize the acidic carbopol [13]. Penetration enhancers are used to enhance and improve the drug permeability across the skin barrier as well as act as the plasticizers in the formulations to promote the drug mobility across skin. In the present study, the main objective is to prepare the suitable gel-type drug reservoir for transdermal patch delivery system. The gel was evaluated by* in vitro* drug release studies and physiochemical tests.

## 2. Materials and Methods

### 2.1. Materials

Galantamine hydrobromide was purchased from Xi'an Yiyang Bio-Tech (Xi'an City, China). Carbopol 940 was bought from Fisher Scientific (Waltham, MA, USA). Triethanolamine, propylene glycol, sodium chloride, potassium chloride, monopotassium phosphate, disodium monohydrogen phosphate, and sodium hydroxide were obtained from Merck (Darmstadt, Germany). Trifluoroacetic acid and acetonitrile were obtained from Avantor Performance Materials (Center Valley, PA, USA). All chemicals were of reagent or analytical grade and were used without further purification.

### 2.2. Formulation of the Gel-Type Drug Reservoir

The gel-type drug reservoir was prepared by dissolving carbopol and galantamine hydrobromide in deionized water at 75°C. Triethanolamine which acts as the neutralizer was added to the drug/polymer mixture. This mixture was stirred until homogenized gel formulation is obtained. Permeation enhancer was then added to the gel formulation. Continuous stirring was performed until the formation of a translucent gel. The final gel formulation was examined so that there were no formation of crystals and no phase separations.

### 2.3. *In Vitro *Drug Release Studies

#### 2.3.1. Experimental Setup

*In vitro* drug release study was carried out by using Franz diffusion cells (PermeGear, V9A) with receptor volume of 20.1 cm^3^ and effective diffusion area of 4.9 cm^2^. The receptor compartment was filled with the phosphate buffer, pH 7.4, which was maintained at 37 ± 0.5°C. The solution was agitated by using a magnetic stirrer. 2 g of gel was then placed in the donor compartment. The cellulose acetate membrane with 0.22 *μ*m pore size was employed as the barrier between the receptor and donor compartment. Synthetic cellulose acetate membrane was used to predict the mechanism of permeation process. This membrane also acts as a quality control tool prior to the usage of biological skins such as human and animal skin [14]. This can reduce the loss of biological samples and also obey the animal ethics.

#### 2.3.2. Drug Release from Galantamine Hydrobromide-Loaded Gels

The drug release from drug-loaded gels was performed by collecting samples (0.5 mL) from the Franz diffusion cells at time intervals of 1, 2, 3, 4, 5, 6, 7, and 8 h. Each time withdrawal of samples will be replenished by 0.5 mL fresh phosphate buffer into the receptor compartment to maintain the same initial volume. The samples taken out from the receptor compartment were then analyzed by using HPLC. Amount of drug permeated was calculated using previous calculated standard calibration curve. The effects of each composition in the formulation on drug release were investigated by changing the percentage of polymer, neutralizer, drug, and enhancer as well as the types of enhancers.

#### 2.3.3. HPLC Analysis

The galantamine hydrobromide analysis was studied by using Waters 1525 HPLC system with Waters 2489 UV/VIS detector. The isocratic HPLC system consists of Agilent Eclipse XDB RF C18 column (5 *μ*m, 4.6 × 150 mm), with trifluoroacetic acid/water/acetonitrile (0.01/85/15 v/v) as the mobile phase. The mobile phase was delivered in the flow rate of 1 mL/min and the wavelength chosen for galantamine hydrobromide detection was 290 nm. The standard calibration curve was plotted in concentration range of 100 ppm to 1000 ppm. The calibration curve showed linear relationship between the peak area and the concentration of galantamine hydrobromide solution, with *R* ^2^ > 0.99.

#### 2.3.4. Statistical Analysis

Data were expressed as mean ± S.D. of three replicates. Statistical analysis was performed by one-way analysis of variance (ANOVA) with *P* < 0.05.

### 2.4. Characterizations of Gel Formulations

#### 2.4.1. Visual Observation

The physical appearance, homogeneity, texture, and stability of the selected gel formulations were studied by visual observations.

#### 2.4.2. pH

In order to test the pH of the formulated gels, the gels were first diluted using deionized water in the dilution factor of 100 (gel : deionized water = 1 : 100). After the suspensions were formed, the pH of each suspension was tested by using pH meter (Mettler Toledo, Delta 320).

#### 2.4.3. Drug Content Analysis

Drug content analysis was used to determine the uniform distribution of galantamine hydrobromide in the gels. The gels were diluted using deionized water with dilution factor of 100 (gel : deionized water = 1 : 100) prior to the drug content analysis. The mixtures were vortexed for 5 mins in order to ensure homogeneous mixing of gels and deionized water. Then, the mixtures were subjected to the centrifugation at 1000 rpm for 20 mins. The drug content of each gel formulation was determined by using HPLC.

## 3. Result and Discussion

### 3.1. *In Vitro* Drug Release Studies

#### 3.1.1. Effects of Carbopol Amount on the Drug Release

**Figure 1:**
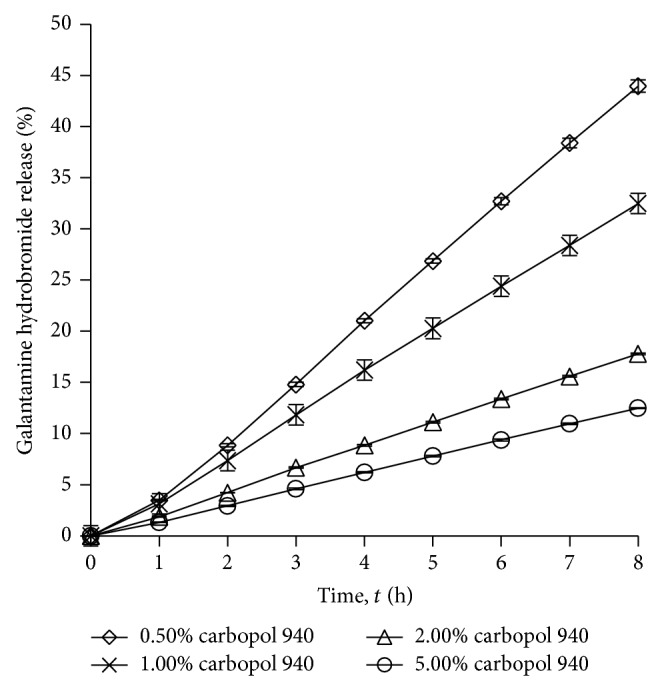
Drug release profile of gel drug reservoirs with different carbopol 940 percentage (mean ± S.D.; *n* = 3).

Figure 1 shows the effect of carbopol amount on the percentage of galantamine hydrobromide release.  Table 2 shows the percentages of drug release at 8 h when changing carbopol concentration in gels (0.50%, 1.00%, 3.00%, and 5.00% w/w). The percentage of galantamine hydrobromide release was inversely proportional to the percentage of carbopol amount. The highest drug release was observed when 0.50% w/w of carbopol was used. The drug release was reduced significantly with increasing the carbopol percentage to 1.00% w/w, 2.00% w/w, and 5.00% w/w. These results were similar to the work done by Batheja et al. [15]. They proposed that this may be due to higher complexity of gel network, produced by higher polymer content. This complex gel network caused longer diffusion pathway of drug to be permeated through the membrane.

#### 3.1.2. Effects of Triethanolamine Amount on the Drug Release

**Figure 2:**
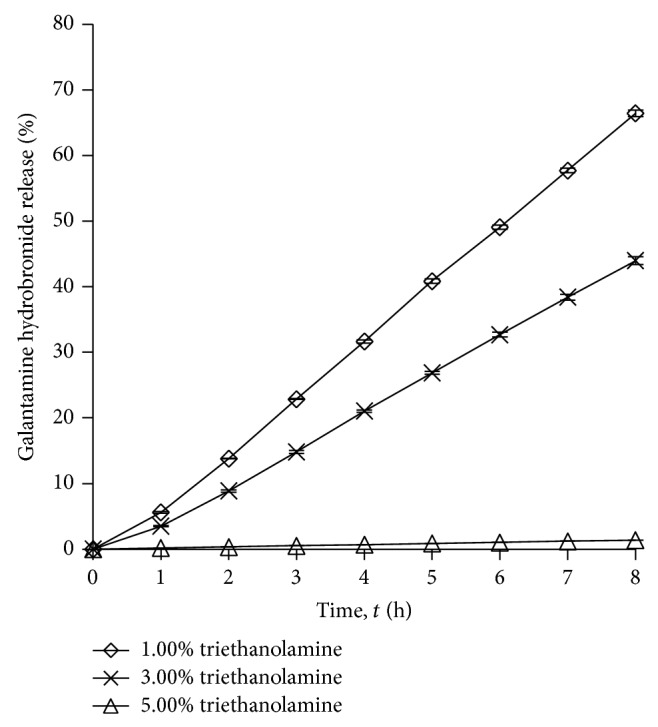
Drug release profile of gel drug reservoirs with different triethanolamine percentage (mean ± S.D.; *n* = 3).

Figure 2 and Table 2 show the effect of the amount of triethanolamine (1.00%, 3.00%, and 5.00% w/w) on the percentage of drug release. When the amount of triethanolamine was increased, the percentage of drug release was reduced. Triethanolamine (1.00% w/w) in the gel caused higher drug release compared to when there was 5.00% w/w of triethanolamine in the gel. The reason could be due to higher amount of triethanolamine causing more viscous and more complex gel formation compared to lower amount of triethanolamine. Thus, the drug molecules are more difficult to be released from the gel [15].

#### 3.1.3. Effects of Galantamine Hydrobromide on Drug Release

**Figure 3:**
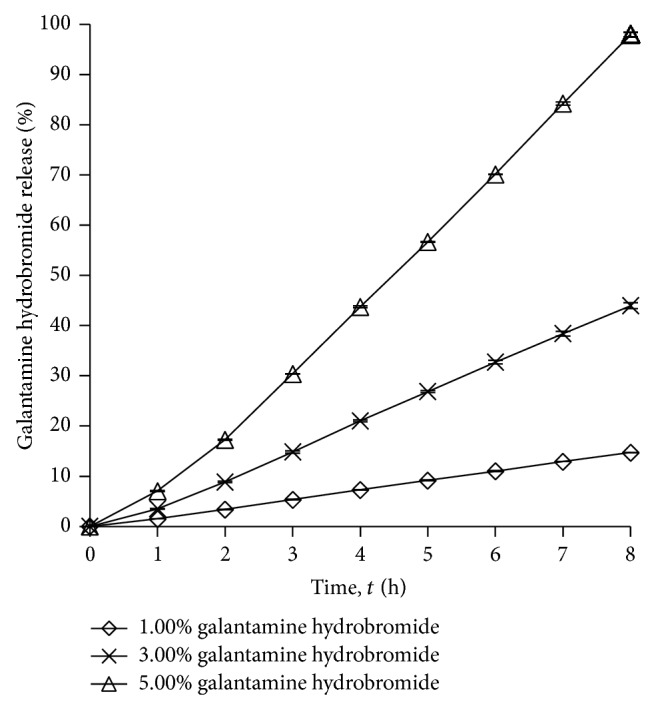
Drug release profile of gel drug reservoirs with different galantamine hydrobromide percentage (mean ± S.D.; *n* = 3).

Figure 3 shows the drug release profile for the gels loaded with 1.00%, 3.00%, and 5.00% w/w of drugs, with fixed amount of other compositions.  Table 2 shows the percentages of drug release at 8 h when different concentration of galantamine hydrobromide was used in the formulations. The galantamine hydrobromide is saturated after 5.00% w/w of drug loading, which the drug crystals observed in gel system. From the results, the percentage of drug release is directly proportional to the drug content in the gel. The highest drug release was observed when 5.00% w/w of galantamine hydrobromide was used. The obtained results obeyed Fick's diffusion law, in which drug permeation was increased when drug concentration increased, with the condition that drug concentration was not saturated in the formulations. Similar results were also reported by Park et al. where the highest drug concentration in formulation produced the highest drug release [16].

#### 3.1.4. Effects of Enhancer Types on Drug Release

**Figure 4:**
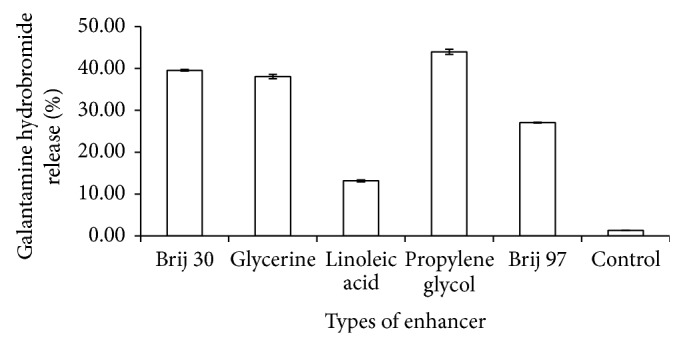
Drug release profile of gel drug reservoirs with different types of enhancer (mean ± S.D.; *n* = 3).

The effects of different types of penetration enhancers on drug release were investigated by changing the enhancers used in gels, namely, Brij 30, glycerine, linoleic acid, propylene glycol, and Brij 97. The enhancer which provides the best enhancement effect was then investigated on the effects of enhancer content on the drug release.  Figure 4 and Table 2 show the effect of the enhancer types on drug release. All the penetration enhancers exhibited significantly higher drug release when compared to the control.  Table 1 also lists the enhancement ratio of each penetration enhancer. Among these penetration enhancers, the highest percentage of drug release (43.95 ± 0.60%) and highest enhancement ratio (32.44) were obtained when propylene glycol is used. Similar results were reported by Gao et al. where propylene glycol has the highest enhancing activities [17]. Propylene glycol can be considered safe due to its low irritancy effect on skin [18]. Thus, the propylene glycol was selected as the penetration enhancers for further studies.

#### 3.1.5. Effects of Enhancer Amount on Drug Release

**Figure 5:**
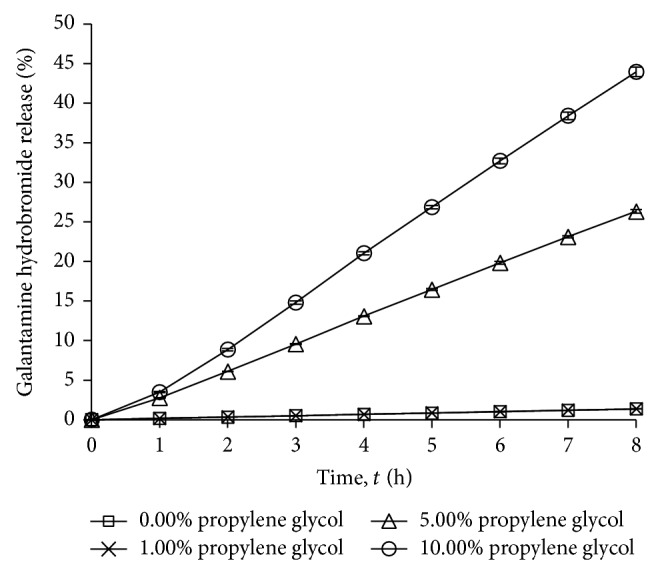
Drug release profile of gel drug reservoirs with different propylene glycol percentage (mean ± S.D.; *n* = 3).

Propylene glycol showed the highest drug release when compared to other penetration enhancers.  Figure 5 and Table 2 show the effects of propylene glycol amount (0.00%, 1.00%, 5.00%, and 10.00% w/w) on drug release. There was no significant improvement on drug permeability when the amount of propylene glycol was increased from 0.00% to 1.00% w/w. However, an improvement in drug release percentage was observed when percentage of propylene glycol was increased from 5.00% to 10.00% w/w. The highest drug release percentage was observed with gel which consisted of 10.00% w/w propylene glycol. Santos et al. and Trottet et al. also deduced that propylene glycol content in the formulations is directly proportional to the amount of drug permeated and drug release [19, 20].

### 3.2. Characterizations of Gel Formulations

Theoretically, gels with higher drug release give higher amount of drug penetration through human skin. Hence, three galantamine hydrobromide-loaded gel formulations (F1, F2, and F3) that provide the highest drug release were selected for further physical studies.  Table 3 shows the composition of these selected gel formulations.

#### 3.2.1. Visual Observation

**Figure 6:**
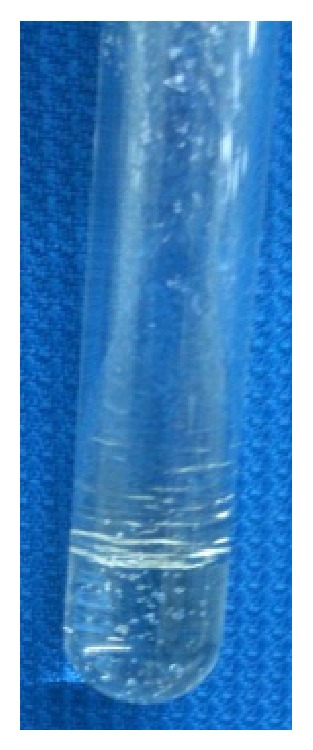
Formulation without drug.

Table 4 shows the physical appearance for galantamine hydrobromide-loaded gel formulations. While Figure 6 shows the gel formulation without drug loading, gel without drug is clear, transparent, and smooth. While F1, F2, and F3 gels are translucent, homogeneous, smooth, and stable, there were no crystals and no phase separation observed in the gel systems. These drug-loaded gels are less viscous and less sticky compared to the formulation without drug.

#### 3.2.2. pH

pH values of F1, F2, and F3 formulations were stated in Table 4. All formulated gels had pH in the range of 7.06 ± 0.01 to 7.80 ± 0.01. This pH range is suitable for skin care and gave no irritancy effect.

#### 3.2.3. Drug Content

Table 4 shows that all gel formulations contained drug content in the range of 85% to 100%. These results suggested that galantamine hydrobromide was uniformly distributed in the carbopol gels and the loss of drug during formulation processes was insignificant.

## 4. Conclusion

The gel drug reservoirs were successfully prepared by adding galantamine hydrobromide in the gels. The* in vitro* release studies showed that the amount of polymer, amount of cross-linker, amount of drug, types of enhancer, and amount of enhancer can affect the drug release from the gel formulations. The highest drug release was observed when formulation had lower amount of polymer, lower amount of cross-linker, higher amount of drug, and higher amount of chemical permeation enhancer. The formulated gel drug reservoirs were translucent and smooth and no formation of crystals or separating layers were observed. pH values of the gels are in range which would not cause skin irritation. High and uniform drug content is observed in all gels. The formulated gels have high drug release, high stability, mild pH, and high drug content, which met the basic criteria as drug reservoirs for transdermal drug delivery system. These gel drug reservoirs thus could be used to fabricate transdermal patch system for the treatment of Alzheimer's disease.

## Supplementary Material

Figure S1 shows the standard calibration curve for galantamine hydrobromide. The curve was plotted in concentration range from 100 ppm to 1000 ppm, with line equation (y = 884.86x - 16100) and correlation coeffiecient (R^2^ = 0.9956).
Figure S2 shows the HPLC spectrum for galantamine hydrobromide with concentration of 100 ppm. The peak retention time is 1.146 mins and area under peak is 79859 μV∗sec.
Figure S3 shows the graphical abstract for this work. The drug release studies were carried out by using Franz Diffusion Cell with three parts, namely donor compartment, barrier and receptor compartment. Donor compartment was filled with galantamine hydrobromide-loaded gel and receptor compartment was filled with phosphate buffer solution. Cellulose acetate membrane was used as the barrier between donor and receptor compartment. The release of galantamine hydrobromide from gel into receptor compartment was analyzed quantitavely by using HPLC.

## Figures and Tables

**Table 1:** Drug release percentage at 8 h and enhancement ratio of each enhancer.

Enhancer types	Drug release percentage at 8** **h (%)	Enhancement ratio
Brij 30	39.54 ± 0.20	29.18
Glycerine	38.04 ± 0.53	28.07
Linoleic acid	13.17 ± 0.24	9.72
Propylene glycol	43.95 ± 0.60	32.44
Brij 97	27.04 ± 0.12	19.96
Control	1.35 ± 0.00	—

**Table 2:** Percentage of drug release at 8 h from different gel formulations (mean ± S.D.; n = 3).

Factors	Weight percentage (% w/w)	Drug release percentage at 8 h (%)
Carbopol amount	0.50	43.95 ± 0.60^*^
	1.00	32.47 ± 0.23
	2.00	17.79 ± 0.08
	5.00	12.48 ± 0.05

Triethanolamine amount	1.00	66.42 ± 0.51
	3.00	43.95 ± 0.60^*^
	5.00	1.37 ± 0.00

Galantamine hydrobromide amount	1.00	14.71 ± 0.04
	3.00	43.95 ± 0.60^*^
	5.00	97.94 ± 0.50

Propylene glycol amount	0.00	1.35 ± 0.00
	1.00	1.37 ± 0.01
	5.00	26.33 ± 0.24
	10.00	43.95 ± 0.60^*^

^*^denotes the repeated formulations, with the same composition.

**Table 3:** Three gel formulations with the highest percentage of drug release.

Formulation code	F1	F2	F3
Carbopol 940 (% w/w)	0.50	0.50	0.50
Triethanolamine (% w/w)	3.00	1.00	3.00
Galantamine hydrobromide (% w/w)	3.00	3.00	5.00
Propylene glycol (% w/w)	10.00	10.00	10.00

**Table 4:** Gels characteristics.

Formulation code	F1	F2	F3
Appearance	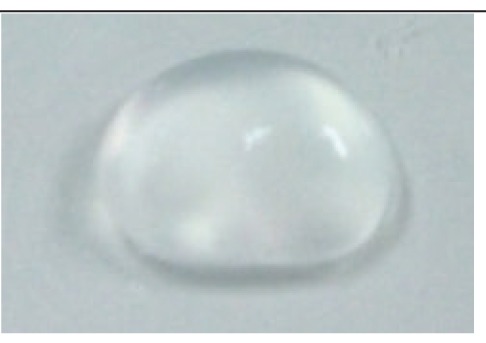	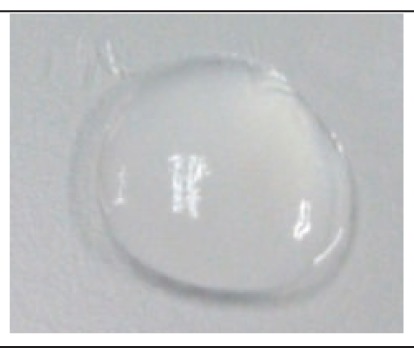	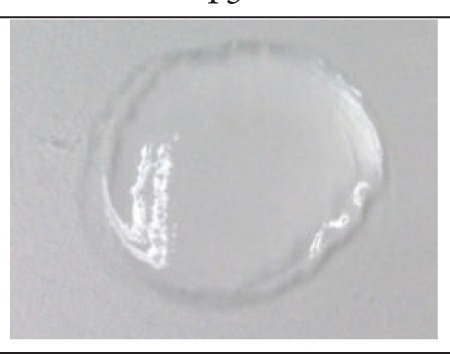

Transparency	Translucent	Translucent	Translucent

Homogeneity	Highly homogeneous (no crystals observed)	Highly homogeneous (no crystals observed)	Highly homogeneous (no crystals observed)

Stability	No phase separation	No phase separation	No phase separation

Texture	Smooth	Smooth	Smooth

pH	7.80 ± 0.01	7.06 ± 0.01	7.45 ± 0.02

Drug content (%)	93.79 ± 1.92	96.67 ± 1.60	95.72 ± 1.81

## Abbreviations

HPLC: High performance liquid chromatography
UV/VIS: Ultraviolet/visible
S.D.: Standard deviation
ANOVA: Analysis of variance.
