# Supplementary material for: Formulation and Evaluation of Galantamine Gel as Drug Reservoir in Transdermal Patch Delivery System
Source: ScientificWorldJournal. 2015 Feb 26;2015:495271. doi: 10.1155/2015/495271 (PMC4374332; doi:10.1155/2015/495271)
Supplement: Supplementary file 1 — Figure S1 shows the standard calibration curve for galantamine hydrobromide. The curve was plotted in concentration range from 100 ppm to 1000 ppm, with line equation (y = 884.86x - 16100) and correlation coeffiecient (R2 = 0.9956). Figure S2 shows the HPLC spectrum for galantamine hydrobromide with concentration of 100 ppm. The peak retention time is 1.146 mins and area under peak is 79859 μV∗sec. Figure S3 shows the graphical abstract for this work. The drug release studies were carried out by using Franz Diffusion Cell with three parts, namely donor compartment, barrier and receptor compartment. Donor compartment was filled with galantamine hydrobromide-loaded gel and receptor compartment was filled with phosphate buffer solution. Cellulose acetate membrane was used as the barrier between donor and receptor compartment. The release of galantamine hydrobromide from gel into receptor compartment was analyzed quantitavely by using HPLC. [file 495271.f1.pdf]

SUPPORTING INFORMATION

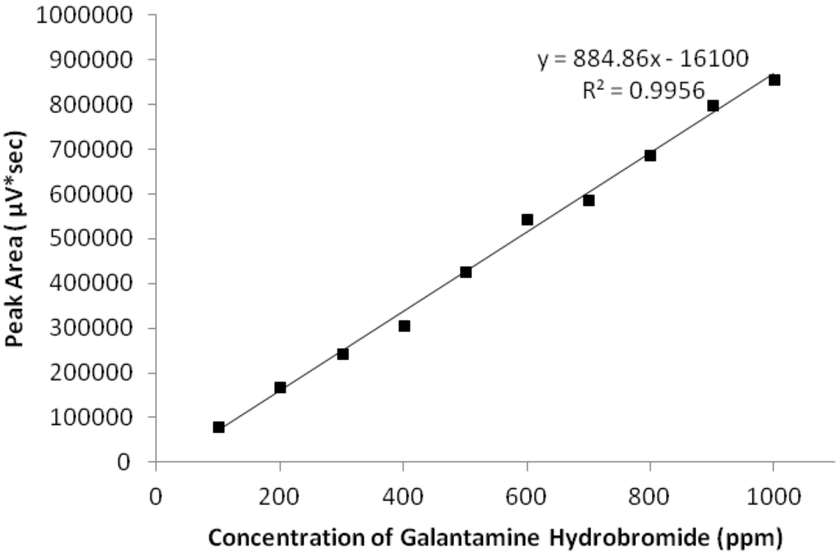

Supplementary Figure S1. Standard Calibration Curve for Galantamine Hydrobromide with line equation (y = 884.86x - 16100) and correlation coeffiecient (R^2 = 0.9956).

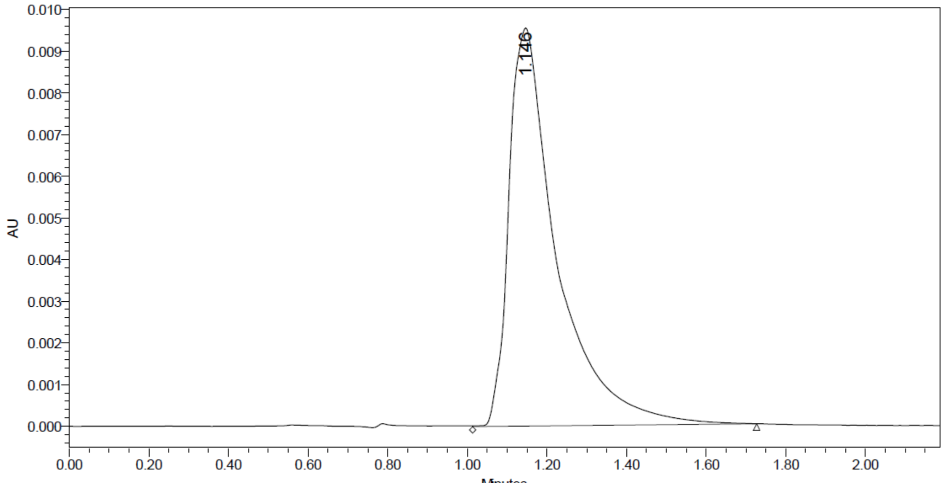

Supplementary Figure S2. HPLC Spectrum for Galantamine Hydrobromide standard solution (100 ppm). The peak observed at retention time of 1.146 mins with area of 79859 µV*sec.

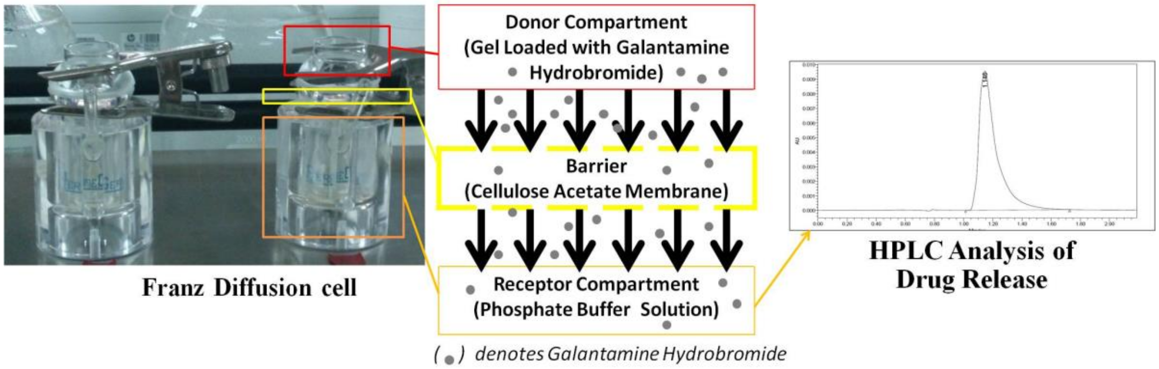

Supplementary Figure S3. Graphical Abstract.
